# Case of Spina Bifida

**Published:** 1852-07

**Authors:** J. P. Russell

**Affiliations:** Waveland Montgomery county, Ill.


ART. IV.—*Case of Spina Bifida.* By J. P. Russell, M.D., of Waveland Montgomery county, Ill.

March, 16, 1851. I was summoned to visit Mrs. F——; arrived at 1 o'clock, A.M., found her in her first labor. On examination a substance of a fleshy feel, as if of the placenta, presented. I could not for some time think it anything else, yet there was no hæmorrhage. The os uteri having dilated well, I passed my hand behind the tumor until my fingers came in contact with the sacrum and lumbar region of the child. This was done immediately after the membranes had given way so as to facilitate the operation of turning, which I was apprehensive would be necessary. With a slight force the breech was made to assume the direction of the pelvic cavity, and as I could not easily succeed in bringing down the feet, I contented myself with the breach position. The labor was strong and severe, which was, however, mitigated by chloroform. This remedy was suggested by T. W. Florer, M.D., a neighboring physician who was called in by my request.

We were now both in doubt in regard to the presenting substance in advance of the child. It was very tense at every pain, having the spongy, fleshy feel before mentioned, and containing a pint or more of fluid. The pains soon increased in force and the tumor gave way and became flaccid. The delivery of a large male child was effected at 3 o'clock, P. M., (still born). The tumor proved to be a spina bifida, situated near the two lower lumbar vertebræ. The central portion of the integuments of the tumor, where it ruptured and gave escape to its contents, was in a disorganized state. The inner surface was smooth, polished, and of a dark red color. Externally it was much congested and had the appearance of a scrotum. (Some insisted that it was a hog's ear; others of the neighborhood, more profound in tracing cause and effect, attributed it to a sight of the elephant, inasmuch as the mother had visited a menagerie some months previous to her confinement.)

